# Intimate partner violence related to disclosure of sickle cell disease during pregnancy: evidence from the sickle cell belt of central India

**DOI:** 10.3389/fgwh.2025.1525168

**Published:** 2025-05-22

**Authors:** Nafisa Halim, Archana Patel, Janet M. Turan, Anuradha V. Shrikhande, Patricia L. Kavanagh, Mari-Lynn Drainoni, David Henderson, Shweta Murali Anand, Nandini Agarwal, Patricia L. Hibberd

**Affiliations:** ^1^ Department of Global Health, Boston University School of Public Health, Boston, MA, United States; ^2^ Lata Medical Research Foundation, Nagpur, India; ^3^ Datta Meghe Institute of Higher Education and Research, Wardha, India; ^4^ Department of Health Policy and Organization of the School of Public Health, University of Alabama at Birmingham, Birmingham, AL, United States; ^5^ Sickle Cell Association of Nagpur, Nagpur, India; ^6^ Department of Pediatrics, Boston Medical Center, Boston University Chobanian & Avedisian School of Medicine, Boston, MA, United States; ^7^ Department of Medicine (Infectious Diseases), Boston Medical Center, Boston University Chobanian & Avedisian School of Medicine, Boston, MA, United States; ^8^ Department of Health Policy and Management, Boston University School of Public Health, Boston, MA, United States; ^9^ Evans Center for Implementation and Improvement Sciences, Boston University Chobanian & Avedisian School of Medicine, Boston, MA, United States; ^10^ Department of Psychiatry, Boston University Chobanian & Avedisian School of Medicine, Boston, MA, United States; ^11^ Division of Psychiatry, Boston Medical Center, Boston, MA, United States; ^12^ Department of Epidemiology, Harvard TH Chan School of Public Health, Boston, MA, United States; ^13^ Institute for Medical Research, Durham, NC, United States

**Keywords:** intimate partner violence, sickle cell disease, sickle cell trait, pregnancy, antenatal screening, stigma and discrimination, stress

## Abstract

**Background:**

Intimate partner violence (IPV) negatively impacts pregnant women and their unborn children. Globally, an estimated 19%, 9%, and 6% of women experience psychological, physical, and sexual IPV, respectively, during pregnancy. These rates are higher among pregnant women living with a stigmatizing disease. In this study, we examined the effect of antenatal screening for sickle cell disease (SCD) using the sickle cell solubility test on the risk of IPV among pregnant women in the city of Nagpur in Maharashtra state of India. We hypothesized that a positive solubility test increases the risk of IPV via partner disclosure.

**Methods:**

We conducted a cohort study comparing IPV in 182 pregnant women, before (baseline) and after (endline) having a solubility test. Of the 182 participants, 91 were pregnant women with a positive solubility test and 91 with a negative solubility test. We used the 49-item Indian Family Violence and Control Scale (*α* = 0.88) to measure IPV and estimated associations using binomial logistic regressions with robust standard errors.

**Results:**

Pregnant women with a positive solubility test were at least twice as likely to experience physical, sexual, or psychological IPV as pregnant women with a negative solubility test, even after adjusting for baseline differences between these two groups on common IPV risk factors including the lower level of education and scheduled-caste membership.

**Conclusion:**

Pregnant women who have a positive solubility test are at risk of IPV after following routine instructions to disclose their test results to their male partners, so that they can undergo further testing to determine the baby’s risk of SCD, sickle cell trait, or no risk.

**Implications:**

In resource-poor settings with high SCD prevalence, antenatal clinics are increasingly screening pregnant women to prevent mother-to-child transmission of SCD. There is a need to integrate strategies for women to disclose sickle cell screening test results and prevention of IPV caused by male partners.

## 1 Introduction

Intimate partner violence (IPV) is not uncommon during pregnancy. Globally, an estimated 19%, 9%, and 6% of women experience psychological, physical, and sexual IPV, respectively, during pregnancy (1). These rates are higher among pregnant women living with diseases that are “stigmatizing,” known as the act of reducing affected individuals “from a whole and usual person to a tainted, discounted one,” undeserving of the same treatment as what is given to unaffected individuals (2–6). In addition to the emotional and psychological devastation it can inflict on pregnant women (7, 8), IPV can influence the course of pregnancy and the risk of adverse childbirth including pregnancy loss, preterm delivery, and maternal or neonatal mortality (9–12). Therefore, understanding the levers of risk of stigmatization of women during pregnancy can inform safer pregnancy via prevention of stigma-induced IPV.

Screening pregnant women for sickle cell disease (SCD) is a standard part of first-trimester antenatal care (ANC) in India (13, 14), home to the second highest number of children born with SCD, approximately 42,000 per year (15, 16), with the majority from the scheduled tribe communities (15, 17). Due to geographic isolation and socioeconomic deprivation, endogamy has remained a prevailing practice of union formation among scheduled tribe communities, potentially contributing to the higher rates of SCD affecting these communities (16, 18). SCD is an inherited group of blood disorders affecting hemoglobin within red blood cells which causes them to assume a sickle shape. The goal of SCD screening is to help pregnant couples understand their risk of having a child with sickle cell anemia (SCA), the most severe form of SCD, based first on maternal and then paternal screening via the sickle cell solubility test and diagnosis of either SCA or sickle cell trait (SCT) via the electrophoresis test of a positive solubility test. SCT is asymptomatic or associated with mild symptoms. Such knowledge aims to aid couples' reproductive decision-making and provide appropriate antenatal care to pregnant women with SCA, who may be unaware of their status until the current pregnancy due to the milder presentation (i.e., fewer pain episodes and less anemia) of the Asian haplotype of SCD in India (13).

IPV can occur as an unintended consequence of mandatory partner disclosure of SCD screening results and consequent partner reaction. To elaborate, antenatal screening for SCD involves administering the sickle cell solubility test to pregnant women, in which a positive solubility test denotes the presence of sickle hemoglobin in a blood sample *without distinguishing between SCA and SCT*. Partner disclosure is mandatory in case of a positive solubility test on maternal SCD screening, since diagnoses of both parents inform the risk of SCA in the child. Upon learning about a positive solubility test, there is a risk that men may react by perpetrating violence against their pregnant wives due to socialization into blaming women for poor child outcomes, misunderstanding a positive solubility test as a confirmation of SCA, which is a stigmatizing disease (19–21).

Globally, the literature on the consequences of SCD disclosure is limited, representing a neglected area of research. This paper intends to address this gap by generating evidence on the potential need for disclosure support associated with antenatal SCD screenings, particularly across India's sickle cell belt region encompassing >67 million people (15). This paper assessed whether SCD screenings can pose a risk for IPV among pregnant women in Nagpur, a city located within this region.

## 2 Methods

### 2.1 Context

**Figure 1:**
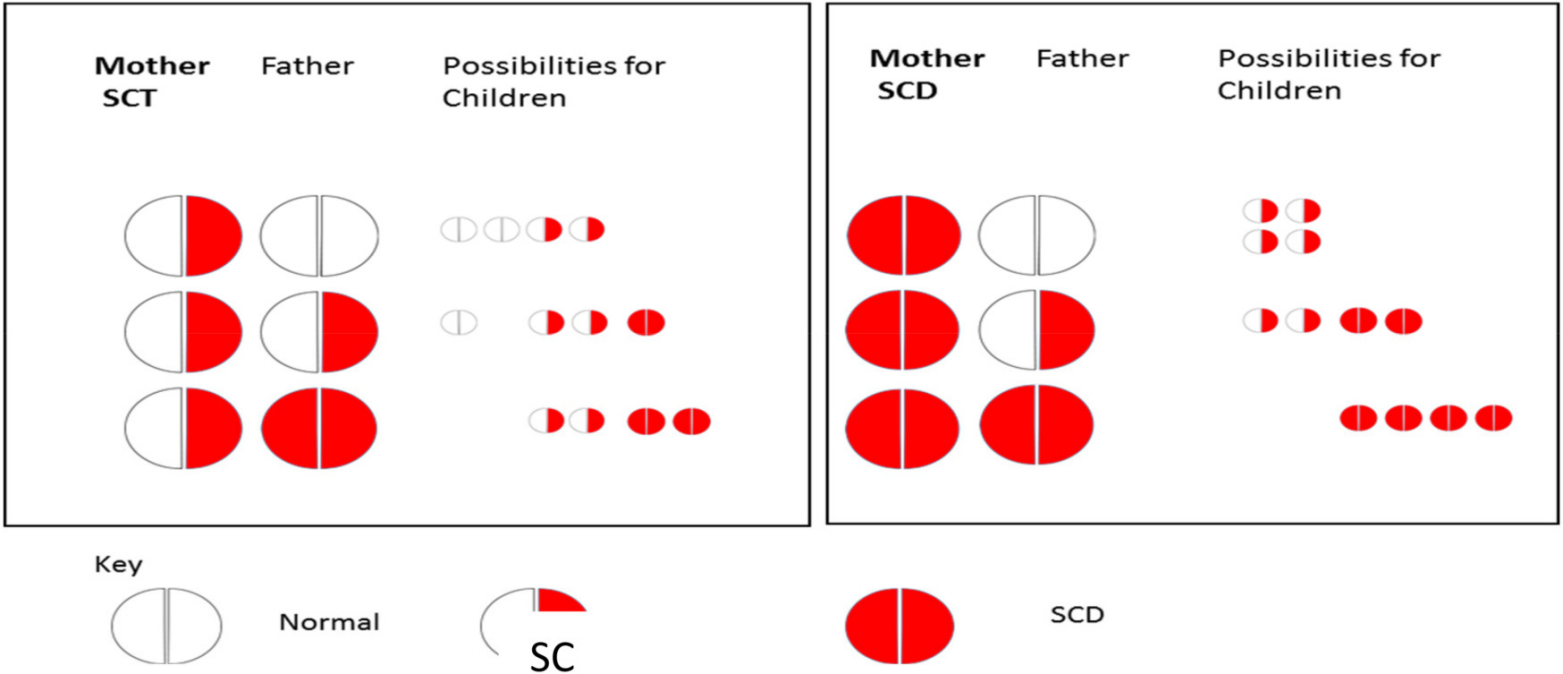
Consequences of a positive solubility test in a pregnant woman.

As a standard part of first-trimester antenatal care (ANC) in India, ANC clinics screen pregnant women for SCD using the sickle cell solubility test and confirm a positive result as either SCA or SCT based on the electrophoresis test. A positive solubility test suggests the presence of at least one copy of HbS, which is indicative of the presence of SCA or SCT. Meanwhile, ANC mandates partner disclosure of a positive solubility test to facilitate paternal screening to determine the risk of the child being born with SCA. Paternal screening is critical in the case of maternal positive solubility test, as pregnant women with SCT or SCA can have children with SCA, depending on whether their male partners have SCT or SCA (see Figure 1). Children born with SCA will inherit two hemoglobin “S” (HbS) genes, one from each parent. Children born with SCT will inherit a HbS gene from one parent and a normal gene from the other parent. Pregnant women with a negative result do not have a risk of giving birth to a child with SCA.

### 2.2 Conceptual framework

Following the socioecological model (22–24), we maintain that SCD stigma occurs at multiple levels of influence, which makes mandatory partner disclosure of SCD screening results a risk factor for IPV among pregnant women. Manifested in micro-interactions (e.g., couple interactions) of the negative beliefs (e.g., possession of undesirable attributes), attitudes (e.g., unworthy of equal status and treatment), and practices (e.g., social exclusion) associated with SCD existing in multiple levels of influence (i.e., society, communities, and institutions), stigma is intended to discriminate or mistreat people (5, 25) including those with SCD (26).

Following the stress process framework (27, 28), we propose ways for how a positive solubility test can trigger a violent response among men in contexts that stigmatize SCD. Lacking knowledge about SCD (29), men may misunderstand a positive solubility test as a confirmation that women have SCA, which is a stigmatizing disease (26). Anticipating having to spend a life with a wife with SCA, men may experience psychological distress as a result. Furthermore, men may worry about the future of the unborn child now having to live with poor health due to SCA or with poor quality of life rife with the experience of discrimination due to prevailing SCA stigma in society (21, 30, 31), leading them to experience psychological distress, and fear of status loss, discrimination, or negative social relations. Consequently, by way of coping with psychological distress (32), men may associate women with a positive solubility test with SCA stereotypes as “damaged” or “no good for childbearing”; accuse women of deceit, when women intentionally hide their SCA result until the current pregnancy; or use other forms of violence, especially if they used IPV in the past, grew up seeing IPV, or currently seeing IPV being perpetrated against women by their male neighbors, friends, or relatives (33–35).

### 2.3 Study setting

We conducted this study in Nagpur, serving scheduled tribe populations living in the sickle cell belt region of India including in Maharashtra and neighboring states. A total of 20 urban primary health centers (UPHCs) served as data collection sites.

### 2.4 Study design

We conducted a cohort study involving pregnant women seeking ANC care in UPHCs in Nagpur in 2019–2020 as the study population. Using interviewer-administered questionnaires, we surveyed 91 pregnant women with a positive solubility test and 91 with a negative solubility test within 3 months of receiving their sickle cell solubility test result about their experiences of IPV 6 months prior to the survey (baseline). A follow-up survey was conducted within 3–4 months after childbirth (endline), where we obtained information on their experiences of IPV since the baseline survey. We implemented this study design to accommodate the participant's availability for this research. Originally, we planned to survey 91 pregnant women with a positive test result and 91 with a negative test result about their experience of IPV 6 months before receiving their sickle cell solubility test results (baseline, prior to partner disclosure) and 6 months after participating in the baseline survey (endline, after partner disclosure). Our study followed the ethics in IPV research, which is to accommodate women's wishes.

Women testing positive participated in the baseline survey within a mean of 1.1 months after receipt of the sickle cell solubility test result, with 95% participating within 3 months, whereas women testing negative did so within a mean of 0.8 months after test results, with 95% participating within 2 months (*p* = 0.003). Women testing positive participated in the endline survey within a mean of 1.5 months after delivery, with 95% participating within 3 months, whereas women testing negative did so within a mean of 2.1 months, with nine women participating in the endline survey after 4 months of delivery inflating the average (*p* = 0.001). Overall attrition between the baseline and endline surveys was 14%, which was higher among women testing positive than that among women testing negative (19.8% vs. 8.8%, respectively; *p* = 0.034). However, within each cohort (women testing positive vs. negative), women who retained in the study and those who dropped out did not differ on baseline IPV or characteristics (results are available upon request).

### 2.5 Sample size

With 91 women per group, we had the power to detect at least a difference of 20 percentage points between two groups in endline IPV rates, assuming a 20% baseline IPV rate among women with a negative test result and a 95% confidence interval. We chose to use a 20% IPV rate, as this was the 12-month prevalence of IPV among a nationally represented sample of Indian women of reproductive age, and to capture the higher rate of IPV experienced pre-pregnancy rather than using the 4% prevalence rate for physical IPV during pregnancy, as using the latter would require a larger sample size (1, 36).

### 2.6 Recruitment

We recruited the first 91 pregnant women with a positive solubility test and 91 with a negative solubility test, from the pregnant women presenting to 1 of 20 UPHCs since the study start date. Information on positive/negative solubility tests was obtained from a UPHC-affiliated laboratory. Women were eligible for this study if they were planning to remain in the study area during pregnancy, labor, and delivery; never attempted or had thoughts of self-harm; had never been treated for mental health issues; were not seeking in-patient care at a medical facility; and had no prior knowledge of their sickle cell status before the current pregnancy.

### 2.7 Data collection

At the baseline, we collected data on common sociodemographic correlates of IPV for the mother (age, age of first marriage, previously married, consanguinity, education, gestational age, religion, caste membership, food insecurity) and the father (age, age at first marriage, education). At the endline, we collected data on partner disclosure and husbands' SCD screening dates and results.

*IPV at the baseline and endline*. We used the 49-item Indian Family Violence and Control Scale (FVCS) (*α* = 0.88) to collect baseline data on experience of physical, sexual, and psychological IPV in the last 6 months prior to the survey and, for endline data, on experience of these types of IPV since participation in the baseline survey.

*Survey administration*. During clinic visits, trained interviewers administered the surveys face-to-face reading out loud questions in Marathi.

### 2.8 Measures

*IPV.* We considered women to have experienced physical IPV if she responded experiencing any one of the following at least once in the last 6 months: *husband slapped her or threw something at her that could hurt her*; *pushed or shoved her*; *hit her with a fist or with something else that could hurt her*; *kicked, dragged, beaten, choked, or burned her*; or *threatened to use or actually used a gun, knife, or other weapon against her*. We used the same method to assess women's experience of sexual IPV (*husband forced her to have sexual intercourse against her will*; *husband forced to do something sexual that she found degrading or humiliating*) and emotional IPV (*husband insulted her or deliberately made her feel bad about herself*; *belittled or humiliated her in front of other people*; *done things to scare or intimidate her on purpose*; *threatened to hurt her*; *hurt people she cares about as a way of hurting her*; *or damaged things that are important to her*).

*Positive/negative solubility test*. We coded women testing positive as 1 and those testing negative as 0.

### 2.9 Statistical analysis.

Aided by our study design, we compared women testing positive with those testing negative for balance on a total of 17 sociodemographic variables using the *t*-test, *χ*^2^ test, or Wilcoxon Mann–Whitney sum test, informing our selection of confounders to adjust in regression models. We found the level of education and caste membership to be different between two groups of women. Religion and father's education were also different, which we ignored in model fitting, since they had no association with IPV in our sample of women (results available upon request). We ran separate binomial logistic regression models to estimate the association between a positive solubility test and risk of any IPV (i.e., physical, sexual, or psychological IPV) at baseline and endline, adjusting for level of education and caste at each time point. We used any IPV to ensure that the outcome variable had enough *yes* vs. *no* events (i.e., IPV occurrences vs. non-occurrences) for model fitting and to avoid overparameterization (i.e., including too many explanatory variables for an outcome with too few events). Endline regression models included baseline IPV as a control with errors corrected for repeated measures using GLM modeling approaches. We fitted models using physical, sexual, and psychological IPV as an outcome, for exploratory purposes (results available upon request).

### 2.10 Ethical clearance

We obtained ethical approval from the Boston Medical Center/Boston University Medical Campus Institutional Review Board in the United States and the Lata Medical Research Foundation in India prior to the study. Women provided signed consent prior to participation.

## 3 Findings

### 3.1 Baseline characteristics and cohort balance

*Women*. The two groups of women (positive and negative solubility tests) were comparable on 7 of 10 characteristics, including age (26.6 years vs. 25.5 years), age at first/current marriage (22.5 years vs. 22.7 years), first-time married (98.9% vs. 96.7%), husband related by blood (14.3% vs. 11.0%), school attendance (97.8% vs. 98.9%), gestation age at SCD screening (19.9 weeks vs. 20.1 weeks), and no food insecurity (96.7% vs. 98.9%). However, the two groups differed on the highest level of education completed, with twice as many women with a negative solubility test as those with positive having completed at least the secondary level of education (90% vs. 42%, *p* < 0.0001). Furthermore, more women with a positive solubility test practiced Buddhism/Neo-Buddhism and were from a scheduled caste or scheduled tribe, compared with those who tested negative (57% vs. 32%, respectively; *p* = 0.04; and 69% vs. 49%, respectively; *p* = 0.01). Most women underwent SCD screening in the second trimester, with 77% of women testing positive undergoing SCD screening at 24 weeks of gestation, compared with 71% of women testing negative (Table 1).

**Table 1:** Baseline characteristics of women and husbands, all and by sickle cell solubility test result, *n* = 182, Nagpur (India), 2019–2020.

Variable			All (*n* = 182)		Women with a positive solubility test (*n* = 91)		Women with a negative solubility test (*n* = 91)		*p*
			Mean/*f*	(%)	Mean/*f*	(%)	Mean/*f*	(%)	
Woman	Age (in years), Mean(SD)		26.0	4.3	26.6	5.1	25.5	3.4	0.08
	Age of first/current marriage		22.6	2.9	22.5	2.9	22.7	2.9	0.68
	Married before	Yes	4	(2.2)	1	(1.1)	3	(3.3)	0.31
		No	178	(97.8)	91	(98.9)	88	(96.7)	
	Husband related to woman	Yes	23	(12.6)	13	(14.3)	10	(11.0)	0.50
		No	159	(87.4)	78	(85.7)	81	(89.0)	
	Ever attended school	Yes	179	(98.4)	89	(97.8)	90	(98.9)	0.56
		No	3	(1.7)	2	(2.2)	1	(1.1)	
	Highest standard completed								
	Lower primary		54	(29.7)	50	(54.9)	4	(4.4)	<0.0001
	Upper primary		8	(4.4)	3	(3.3)	5	(5.5)	
	Secondary		60	(33.0)	18	(19.8)	42	(46.2)	
	Undergraduate		51	(28.0)	18	(19.8)	33	(36.3)	
	Graduate		9	(5.0)	2	(2.2)	7	(7.7)	
	Gestational age at SCD screening (in weeks), mean (SD)		20.0	(7.9)	19.9	(8.1)	20.1	(7.8)	0.89
	Religion								
	Hindu		79	(43.4)	32	(35.2)	47	(51.7)	0.00
	Muslim		13	(7.1)	3	(3.3)	10	(11.0)	
	Buddhist/Neo-Buddhist		81	(44.5)	52	(57.1)	29	(31.9)	
	Other		9	(5.0)	4	(4.4)	5	(5.5)	
	Caste membership								
	Scheduled caste or scheduled tribe (SCST)		108	(59.3)	63	(69.2)	45	(49.5)	0.01
	Other backward caste		38	(20.9)	18	(19.8)	20	(22.0)	
	Not SCST or OBC		36	(19.8)	10	(11.0)	26	(28.6)	
	Household experienced food shortages this year								
	Food shortage for any number of months	Yes	4	(2.2)	3	(3.3)	1	(1.1)	0.31
		No	178	(97.8)	88	(96.7)	90	(98.9)	
	Food shortage between 1 and 6 months	Yes	4	(97.8)	3	(3.3)	1	(1.1)	0.31
		No	178	(2.2)	88	(96.7)	90	(98.9)	
	Household experienced food shortage in the last 4 weeks	Yes	1	(0.6)	0	(0.0)	1	(1.1)	0.32
		No	181	(99.5)	91	(100.0)	90	(98.9)	
	Disclosed the positive solubility test to husbands (*n* = 68)*	Yes			65	(96.0)			
		No			3	(4.0)			
Man	Age (in years), mean (SD)		30.8	(4.8)	31.5	(5.5)	30.2	(3.9)	0.08
	Age of first/current marriage, mean (SD)		28.2	(3.4)	28.1	(3.3)	28.3	(3.4)	0.72
	Ever attended school	Yes	177	(97.3)	87	(95.6)	91	(98.9)	0.17
		No	5	(2.8)	4	(4.4)	1.00	(1.1)	
	Highest standard completed								
	Lower primary		54	(29.7)	50	(55.0)	4	(4.4)	<0.0001
	Upper primary		16	(8.8)	1	(1.1)	15	(16.5)	
	Secondary		79	(43.4)	30	(33.0)	49	(53.8)	
	Undergraduate		30	(16.5)	10	(11.0)	20	(22.0)	
	Graduate		3	(1.7)	0	(0.0)	3	(3.3)	
	Endline data on whether screened for SCD are available (*n* = 73)*	Yes			32	(44.0)			
		No			41	(56.0)			
	SCD screening occurred before the baseline survey (*n* = 32)*	Yes			32	(100.0)			
		No			0	(0.0)			
Design issues	Number of primary health care clinics involved in the study, mean (SD)		20	–	20	–	10	–	NA
	Number of women interviewed per clinic, mean (SD)		9.1	(10.1)	4.6	(3.9)	9.1	(8.7)	0.06
	Number of months between receipt of solubility test result and participation in baseline survey, mean (SD)		1.0	(0.8)	1.1	(0.1)	0.8	(0.1)	0.00

* Results based on the endline survey.

*Men*. In the two groups (men married to women testing positive vs. negative), characteristics of fathers were comparable on three of four characteristics, including age (31.5 years vs. 30.2 years), age at first/current marriage (28.1 years vs. 28.3), and school attendance (95.6% vs. 98.9%). Similar to women, men married to women testing positive or negative completed different levels of education, with male partners of women with a negative solubility test tending to be more educated than those with a positive solubility test (secondary level completed 79% vs. 44%, respectively, *p* < 0.0001).

*Physical, psychological, sexual, and any IPV*. Significantly more women with a positive result vs. those with a negative result reported experiencing physical IPV (20.9% vs. 6.6%, respectively, *p* = 0.005; see Table 2), emotional IPV (31.8% vs. 16.5%, respectively; *p* = 0.015), and any IPV (39.6% vs. 17.6%, respectively; *p* = 0.001) in the prior 6 months at baseline. However, at endline, we found no difference in the rates of physical IPV, emotional IPV, or any IPV between groups.

**Table 2:** Percent of pregnant women experiencing physical, psychological, and sexual IPV by sickle cell solubility test result at the baseline and the endline, Nagpur, 2019–2020.

Variable	Baseline							Endline						
	All (*n* = 182)		Women with a positive test result (*n* = 91)		Women with a negative test result (*n* = 91)		*p*	All (*n* = 156)		Women with a positive test result (*n* = 73)		Women with a negative test result (*n* = 83)		*p*
	*f*	%	*f*	%	*f*	%		*f*	%	*f*	%	*f*	%	
Physical IPV	25	(13.74)	19	(20.88)	6	(6.59)	0.005	20	(12.82)	8	(10.96)	12	(14.46)	0.633
Psychological IPV	44	(24.18)	29	(31.87)	15	(16.48)	0.015	34	(21.79)	19	(26.03)	15	(18.07)	0.249
Sexual IPV	7	(3.85)	5	(5.49)	2	(2.20)	0.248	2	(1.28)	1	(1.37)	1	(1.20)	1.000
Any IPV	52	(28.57)	36	(39.56)	16	(17.58)	0.001	41	(26.28)	21	(28.77)	20	(24.10)	0.586

### 3.2 Regression results

*Baseline*. Compared to women testing negative, those with a positive test result were three times as likely to report having experienced any IPV at baseline (OR: 3.07, *p* = 0.001) in both the unadjusted and adjusted models (Models 1 and 2, respectively; see Table 3). However, neither women's lower level of education nor scheduled caste membership was associated with their risk of IPV at baseline (Model 2).

**Table 3:** Logistic regression estimates of associations between women's sickle cell solubility test result and IPV at the baseline and the endline, Nagpur, 2019–2020.

Variable	Baseline (*n* = 182)						Endline (*n* = 156)					
	Model 1			Model 2			Model 3			Model 4		
	Physical, psychological, or sexual IPV			Physical, psychological, or sexual IPV			Physical, psychological, or sexual IPV			Physical, psychological, or sexual IPV		
	OR	(95% CI)	*p*	OR	(95% CI)	*p*	OR	(95% CI)	*p*	OR	(95% CI)	*p*
Women with a positive test result (Ref.: women with a negative test result)	3.07	(1.55, 6.09)	0.001	2.96	(1.45, 6.02)	0.003	1.27	(0.62, 2.60)	0.510	1.04	(0.47, 2.31)	0.920
Completed primary or secondary education (Ref.: at least undergraduate education)				0.86	(0.41, 1.80)	0.694				0.71	(0.32, 1.56)	0.393
Scheduled caste/scheduled tribe membership (Ref.: not a scheduled caste/scheduled tribe or OBC)				1.50	(0.73, 3.07)	0.269				1.57	(0.72, 3.41)	0.259
Women reporting physical, psychological, or sexual IPV at baseline (Ref.: no reported IPV)										2.44	(1.11, 5.36)	0.026
Constant	0.21	(0.12, 0.37)	0.000	0.19	(0.09, 0.41)	0.000	0.32	(0.19, 0.52)	0.000	0.25	(0.11, 0.57)	0.001
Area under the ROC curve	0.63			0.66			0.53			0.63		

*Endline*. Compared to women with a negative test result, those testing positive were not more at risk for IPV at endline in the unadjusted or adjusted models (Models 3 and 4).

### 3.3 Additional analysis

*Plausibility analysis*. For baseline IPV, the recall period (i.e., 6 months before the baseline survey) includes up to 4 months post-partner disclosure of a positive test result. Ninety-six percent of women testing positive reported partner disclosure, and partner disclosure occurred prior to the baseline survey for 100% of the women for whom such data are available. To elaborate, we know the dates when women underwent the sickle cell solubility tests and when they participated in the baseline survey, and we know when husbands were screened for SCD for nearly 50% of the women for whom such data are available. Based on these dates, we were able to confirm for the women from whom data are available, that 100% of them disclosed their positive solubility test to their husbands prior to the baseline survey.

*Robustness checks.* Additional analyses were performed to repudiate the idea that the observed association between a positive test result and risk for IPV is a proxy of the association between the lower level of education and IPV or between scheduled-caste membership and IPV. While women testing positive mostly had a primary level of education and were members of a scheduled caste (Model 1; see Table 4), neither the lower level of education nor scheduled-caste membership is a risk factor for baseline or endline IPV for our sample of women (Models 2 and 3, respectively). Therefore, a positive association between a positive test result and risk for IPV in the model adjusted for the lower level of education and scheduled-caste membership is suggestive of a direct effect of a positive test result on IPV, independent of the effects of the lower level of education and schedule-caste membership.

**Table 4:** Logistic regression estimates of associations of education and caste membership with a positive solubility test result, and with IPV at baseline and endline, Nagpur, 2019–2020.

Variable	Baseline (*n* = 182)			Baseline (*n* = 182)			Endline (*n* = 156)		
	Model 1			Model 2			Model 3		
	A positive test result			Physical, psychological, or sexual IPV			Physical, psychological, or sexual IPV		
	OR	(95% CI)	*p*	OR	(95% CI)	*p*	OR	(95% CI)	*p*
Women completed primary or secondary education (Ref.: at least undergraduate education)	2.81	(1.45, 5.44)	0.002	1.13	(0.56, 2.26)	0.736	0.75	(0.35, 1.58)	0.444
Women belong to a scheduled caste/scheduled tribe (Ref.: not a scheduled caste/scheduled tribe or OBC)	2.32	(1.24, 4.35)	0.008	1.81	(0.91, 3.59)	0.092	1.75	(0.82, 3.74)	0.152
Constant	0.30	(0.15, 0.59)	0.001	0.25	(0.13, 0.51)	0.000	0.30	(0.15, 0.64)	0.002
Area under the ROC curve	0.66			0.57			0.58		

## 4 Discussion

In this study, we reported two key findings. *First*, consistent with our conceptual framework, we found that pregnant women with a positive result were at significantly increased risk of IPV around the time of screening for SCD. Partner disclosure is a plausible mediator, as per data on whether and when partner disclosure occurred. Ninety-nine percent of women reported having informed their husbands about their positive results, and among all (100%) women for whom data were available, partner disclosure occurred before the baseline survey. The timing of partner disclosure was estimated based on the order when the baseline survey occurred and husbands underwent an SCD screening (indicating husbands' knowledge about their wives' positive test results).

Notably, for reporting this key finding (i.e., a positive result is positively associated with risk of IPV), our reliance on baseline data and not the endline is reasonable in light of the timing of implementation of the baseline and endline surveys. Women testing positive had up to 4 months to have experienced the consequence (i.e., IPV) of partner disclosure by the time they participated in the baseline survey, making the baseline survey offers the more appropriate source of data for this study than the endline survey. Consistently, we did not find women testing positive to be at any greater risk for IPV in the endline, compared with women testing negative. Still, it is important to acknowledge the recall period referred to in the endline survey as a potential confounder. The endline survey asked women about their experience of IPV in the months since their participation in the baseline survey. In all likelihood, these months coincided with the third trimester of pregnancy and the postpartum period, potentially serving as a protection, strong enough to offset the risk of IPV from a positive test result. Furthermore, the observed risk of IPV was likely due to a positive test result, and not entirely due to pre-existing socioeconomic attributes, as suggested by findings that neither the lower level of education nor the scheduled-caste membership had an association with IPV in the baseline or the endline model.

While novel in the context of SCD, this finding is consistent with findings from a long line of research on the disclosure of an HIV-positive status among pregnant women across sub-Saharan African countries. Most sub-Saharan African countries have adopted the WHO recommendation of routine HIV testing during ANC for the prevention of mother-to-child transmission of HIV (37, 38). However, women face risk for IPV as a result of partner disclosure of an HIV-positive status due to HIV stigma (39–41), inhibiting women from getting tested or remaining in care (42–44).

*Second*, we found the presence of higher rates of physical IPV during pregnancy than currently reported. The baseline (14%) or endline (13%) rates of physical IPV were considerably higher than the 4% reported for physical IPV in the 2015–2016 NFHS report (36). Methodological differences between the NFHS and the current study may account for some differences in IPV rates. As opposed to the 2015–2016 NFHS reporting physical IPV among the nationally representative population of pregnant women, we reported physical IPV among a selected sample of pregnant women living in urban areas of the city of Nagpur, Maharashtra. Furthermore, the NFHS used an abbreviated version of the WHO tool for data on experience of physical IPV during pregnancy, whereas we used the Indian Family Violence and Control Scale (FVCS), which involves an extended list of physical IPV acts (45, 46), such as experience with excessive work, which the WHO tool does not include. However, we decided to use the FVCS tool as it captures aspects of IPV that are unique to the Indian context and culture (46).

### 4.1 Strengths and limitations

To our knowledge, this is the first study to examine the association between antenatal screening for SCD and IPV, and one of only a handful of studies on IPV among women with SCD, globally. Nonetheless, our findings must be interpreted within the context of the limitations below. First, due to how the study had to be implemented, we ended up not having “true” baseline rates of IPV prior to SCD screening, affecting our ability to draw more conclusive findings on the relationship between disclosure of SCD screening results and IPV. Originally, we designed the study to survey 91 pregnant women with a positive test result and 91 with a negative test result about their experience of IPV 6 months before (baseline) receiving the sickle cell solubility test results (prior to partner disclosure) and since participation in the baseline survey (endline) (after partner disclosure). With this design implemented, we would have been able to attribute endline differences in IPV between women testing positive and negative to partner disclosure of a positive result, all else being equal at baseline including IPV. However, during study implementation, we had to deviate from the original study design to accommodate women's wishes regarding the timing of their participation in the surveys as per the ethical conduct of IPV research. In theory, to obtain true baseline IPV rates, we could have recruited only those women agreeing to participate in the baseline survey on the same day that they received a test result; however, doing so would have elongated the time of sample recruitment, which could not be supported by the study budget. Second, we relied on women's self-report for data on women's experience of IPV over some time in the past, which can be affected by underreporting due to IPV stigma or recall bias (47). Furthermore, women may be reluctant to disclose IPV in face-to-face interviews due to shame or reluctance to disclose family matters to strangers, leading to underreporting of IPV events. Third, self-selection is a potential source of bias in cohort studies and may lead to over- or underestimation of effects. However, in our study, self-selection is a bias if pregnant women who tested positive had a different set of motivations for study participation compared with those testing negative. Since we did not collect data on motivations informing study participation, we are limited in our ability to confirm or refute if self-selection is indeed a bias of this study. Fourth, we do not know whether and to what extent participants ended up not consenting to participate in the study even when they were deemed eligible. Fifth, since we designed the study for hypothesis testing, we were not able to explore the extent to which the observed association followed the pathways and the contexts articulated in our conceptual framework (see Conceptual framework section). Sixth, we lack data on prior disabilities, which is a risk factor for IPV and, on prior knowledge about SCD, a protective factor. Finally, our study was underpowered to detect a significant difference in IPV rates between women testing positive vs. negative due to our use of 20% for baseline physical IPV during pregnancy, as opposed to the 4% prevailing in India. Using a 4% rate would require too large a sample for a pioneering study such as ours.

Nonetheless, our present evidence of a positive association between a positive test result and risk for IPV, potentially via partner disclosure, remains intact even when we adjust for an array of confounders including the lower level of education and scheduled-caste membership. Future studies should examine potential mechanisms using a mixed-methods design, as well as sources of variation in the risk for IPV among women who test positive on the sickle cell solubility test.

## 5 Conclusions

Pregnancy is revered cross-culturally, for it represents a period of celebration, abundance, and transition. In many cultures, pregnancy elevates women's status in society. Still, pregnancy can represent a period of distress if the pregnancy care practices involve screening for conditions that are stigmatizing. In resource-poor settings, with the advent of inexpensive point-of-care screening technologies, more and more antenatal clinics are screening pregnant women for hereditary diseases for the prevention or early detection of such diseases, as per local (i.e., the state) or global (i.e., the WHO) recommendations. The current study addressed one such technology, sickle cell solubility test to screen for the presence of sickle cell anemia or sickle cell trait in the blood sample. Considering the current findings, there is a need for provider training to integrate disclosure support services focused on IPV prevention into the SCD screening protocol in Nagpur, India. Currently, there is no intervention supporting the safe disclosure of a positive result in India (48). This is concerning considering that the use of the sickle cell solubility test will remain the standard of care for antenatal screening for SCD in primary care settings since the test is not only inexpensive and rapid but also sensitive and specific. Furthermore, the Sickle SCAN and the HemoTypeSC are among the other rather inexpensive point-of-care techniques (paper-based hemoglobin solubility, lateral flow immunoassays, micro-engineered HE, density-based separation, and smartphone-based application tests) that exist globally. Nonetheless, the Sickle SCAN and the HemoTypeSC are yet to be tested for sensitivity and specificity in the context of India, suggesting the use of the sickle cell solubility test for antenatal screening for SCD in India in the foreseeable future.

Fortunately, disclosure support interventions exist in the context of other stigmatizing diseases such as HIV. Jamii Bora is one such support intervention (44), developed using the Interdependence Model of Couples Communal Coping principles, enabling couples to reframe the diagnosis of a disease as a family matter, leading to “communal coping” via joint communication and health decision-making (49, 50) and to a likely reduction in men's perpetration of IPV against women with HIV. Therefore, adapting such interventions for SCD disclosure may improve safety among pregnant women undergoing SCD screenings in antenatal clinics in the sickle cell belt of Central India.

## Data Availability

The raw data supporting the conclusions of this article will be made available by the authors, without undue reservation.
